# Cigarette Smoking and Nicotine Dependence Among Dental Students in Riyadh, Saudi Arabia: A Cross-Sectional Study

**DOI:** 10.7759/cureus.48676

**Published:** 2023-11-11

**Authors:** Sanjeev B Khanagar, Abdullah S Almansour, Hamzah M Alshanqiti, Nawaf F Alkathiri, Mohammed A Asseery, Saud M Altheyabi, Darshan Devang Divakar

**Affiliations:** 1 Department of Preventive Dental Science, College of Dentistry, King Saud Bin Abdulaziz University for Health Sciences, Riyadh, SAU; 2 Research, King Abdullah International Medical Research Centre, Ministry of National Guard Health Affairs, Riyadh, SAU; 3 College of Public Health, Texila American University, Georgetown, GUY; 4 College of Dentistry, King Saud Bin Abdulaziz University for Health Sciences, Riyadh, SAU; 5 Dental Biomaterials Research Chair, Dental Health Department, College of Applied Medical Sciences, King Saud University, Riyadh, SAU

**Keywords:** prevalence, dental education, nicotine dependence, undergraduate students, smoking, cigarette

## Abstract

Introduction: Dental professionals have a vital role in promoting tobacco cessation interventions in dental care settings, and despite being aware of the detrimental effects of tobacco products on individuals’ health, they are still engaged in using tobacco products.

Aim: The aim of this study was to assess the prevalence of cigarette smoking and the level of physical and social nicotine dependence among undergraduate dental students in Riyadh, Saudi Arabia, using standardized tools to develop appropriate tobacco cessation interventions.

Methodology: Dental students from six dental colleges located in Riyadh, Saudi Arabia, were invited to participate in this study. A total of 430 dental students agreed to participate by providing written informed consent. Physical dependence on nicotine was assessed using the Fagerstrom Test for Nicotine Dependence (FTND), and social dependency was assessed using the Kano Test Social Nicotine Dependence (KTSND).

Results: Among the 430 participants, 120 (27%) reported smoking. The level of nicotine dependence assessed for the 120 (27%) participants who had reported smoking using FTND showed 24 (20%) with high nicotine dependence and 18 (15%) with very high nicotine dependence. When compared between the genders, five (16%) of the female participants displayed high nicotine dependency, and two (6%) displayed very high nicotine dependency. Among the male participants, 19 (21%) displayed high nicotine dependency, and 16 (18%) displayed very high nicotine dependency. The study participants also displayed high KTSND scores, male participants with a mean score of 18.20 and female participants with a mean score of 17.97.

Conclusion: Dental students, despite being the facilitators in tobacco prevention and cessation programs, have displayed a high prevalence of cigarette smoking and nicotine dependence. These findings should be considered for designing specific tobacco cessation programs for dental students, along with effective stress management programs. Emphasis should be placed on developing and implementing policies for creating supportive environments in educational institutions. Dental students should be trained and engaged in tobacco prevention and cessation intervention programs for their patients.

## Introduction

Tobacco and its products are considered a major public health problem, and according to the World Health Organization, tobacco kills more than seven million smokers every year [1]. Among the different varieties of tobacco products, cigarette smoking is the most popular and common method of tobacco usage. Cigarette is a highly efficient drug delivery system that can deliver an average of 1-2 mg of nicotine per cigarette [2]. Most smokers are addicted to cigarettes because of their primary addictive component nicotine. Immediately after smoking a tobacco product, nicotine present in the tobacco is absorbed through the mucous membrane and reaches blood levels. Later, it binds to the nicotinic acetylcholinergic (nAch) receptors located in the brain and leads to further stimulation of the adrenal glands causing an immediate release of (epinephrine) adrenaline [3]. This rush of adrenaline in the body causes a “Buzz/Kick” effect by increasing blood pressure, respiration, and heart rate [2]. Nicotine has substantial detrimental effects on the health of tobacco users, and evidence suggests that it contributes to 40-45% of cancer-related deaths, 35% of cardiovascular disease-related deaths, 90-95% of lung cancer-related deaths, 85% of oral cancer deaths, and 75% of deaths caused by chronic obstructive lung disease in industrialized countries [4]. Exposure to nicotine during pregnancy is associated with congenital malformations such as cleft lip and palate [4]. It also has major negative effects on the oral cavity. Evidence suggests that there is an established link between nicotine and oral diseases such as periodontal diseases, candidiasis, precancerous lesions, dry socket, and xerostomia [5].

Despite being aware of the detrimental effects of tobacco products on individuals' health, the prevalence of tobacco usage among medical students varies from 3% in the United States, 18% in France, 19% in Saudi Arabia, 24% in India, 28.4% in Turkey, and 53% in Japan [6-10]. Studies conducted among dental students have reported smoking among 52% of dental students in India, 4.8% in Australia, and 17-22% in Saudi Arabia [11-14].

Dental professionals play a key role in tobacco cessation interventions as they are in constant touch with the patients and are usually the first to evaluate the effects of tobacco on the oral cavity [15]. Dental education institutions worldwide have policies to encourage students to introduce tobacco cessation programs to their patients [16]. Studies have also reported on the willingness of smokers to quit if their dentists recommend it to them [5]. Tobacco cessation interventions have shown varying degrees of effectiveness in dental care settings [17,18]. Considering the vital role of dental professionals in tobacco cessation interventions, it is very important for them to be ideal role models within their societies. Hence, there is a need to assess the level of nicotine dependence among dental students using standardized tools in order to develop appropriate tobacco cessation interventions. Tobacco control laws in Saudi Arabia permit only the sale of a smoking form of tobacco (combustible cigarette) and prohibit the sale of smokeless tobacco products; hence, in this study, we intend to report on combustible cigarette smoking [19]. The data on smoking among the Saudi Arabian population show that 12.1% of its population currently smokes tobacco, with a prevalence of 23.7% among males and 1.5% among females. The data also show that 29.7% of them initiated smoking before the age of 15 years and 60.9% started smoking before the age of 18 years [20]. The previously reported studies conducted among dental students in Saudi Arabia have reported only the prevalence of tobacco usage. Hence, there is a need to assess the level of nicotine dependence among dental students, which can help in planning smoking cessation interventions and counseling programs. The finding of this study can also help in developing/revising dental curricula that can focus on emphasizing the importance of training and engaging dental students in tobacco prevention and cessation interventions for their patients and for planning and implementing policies that can help dental students refrain from the use of tobacco usage, as well as for creating supportive environments in educational institutions. Hence, the aim of this study was to assess nicotine dependence among undergraduate dental students in Riyadh, Saudi Arabia.

## Materials and methods

Study design

A cross-sectional analytical study was conducted among undergraduate dental students in Riyadh, Saudi Arabia. Before initiating the data collection for this study, a research protocol was submitted to the Institutional Review Board, King Abdullah International Medical Research Center (KAIMRC), Riyadh, Saudi Arabia. The research protocol was approved, and an ethical clearance was obtained (Ref. No. IRBC/1547/22).

Sample size and sampling frame

Based on the results of the previous studies reported in the literature [3], the sample size required for this study was estimated to have a power of 80% and a 95% confidence interval for a prevalence of 50% [21,22]. There is an annual variation in student enrollment among health universities in Saudi Arabia. Hence, the number of students enrolled in the dental colleges located in Riyadh, Saudi Arabia, is not uniform. In order to overcome this limitation, an infinite sample was considered, based on which the estimated sample required for this study was 385.

There are six dental colleges in Riyadh, Saudi Arabia; three are private (Alfarabi College, Riyadh Elm University, Dar Al Uloom University), and three are within governmental institutions (King Saud bin Abdulaziz University for Health Sciences, King Saud University, Princess Nora bint Abdulrahman University). Dental students from all the six colleges were requested to participate in this survey. A simple random sampling technique was considered for selecting a minimum of 65 participants to fulfill the quota from each university upon fulfilling the eligibility criteria. However, during the sampling process, only those participants present on the day of sample selection at the institutions were considered. Hence, this may have contributed to the sampling error.

Eligibility criteria

In this study, dental students studying in dental colleges located in Riyadh, Saudi Arabia, who had smoked more than 100 cigarettes and agreed to participate in this study by providing written informed consent, were included. A person who has smoked more than 100 combustible cigarettes in his lifetime and who currently smokes cigarettes is considered a smoker [23]. Dental students who were nonsmokers (based on the Global Adult Tobacco Survey (GATS) criteria that classify nonsmokers as former daily smokers, never daily smokers, former occasional smokers, or never smokers) [24] or those under medication for any psychiatric disorders were excluded from this study. The study participants were informed that no personal identifiers or identity-revealing information would be collected during the process and that their privacy and confidentiality would be protected.

Data collection

Participation in this study was voluntary and anonymous. A structured close-ended questionnaire that was developed in the English language was used for data collection. The first section of the questionnaire comprised demographic details and smoking habits, followed by the second section, which comprised questions related to nicotine dependence using the standardized pre-validated Fagerstrom Test for Nicotine Dependence (FTND) [25,26]. The FTND test provides an ordinal measure of nicotine dependence related to cigarette smoking. It consists of six items that evaluate the quantity of cigarette consumption, the compulsion to use, and dependence. In FTND, the scoring is in the form of yes/no items that are scored from 0 to 1, and multiple-choice items are scored from 0 to 3. The items are summed to yield a total score, ranging from 0 to 10. Dependence scores ranging within 0-2 are considered very low, 3-4 are regarded as low, 5 as moderate, 6-7 as high, and 8-10 as very high dependence. A higher total Fagerstrom score indicates a more intense physical dependence on nicotine.

The third section comprised a standardized pre-validated Kano Test Social Nicotine Dependence (KTSND) [27,28]. The KTSND is used to assess the psychological and psychosocial aspects of smoking. The responses were measured on a four-point Likert scale from "strongly agree," "somewhat agree," "somewhat disagree" to "strongly disagree" with scores of 0, 1, 2, and 3, respectively. The possible range of KTSND scores is 0-30, with 0 indicating low social nicotine dependence and 30 indicating high dependence. Two studies have reported on the metric properties of this KTSND tool, where Cronbach’s alpha coefficient for this scale were 0.82 and 0.89, respectively, indicating a good degree of reliability and generalizability [27,29].

Method of data collection

Data were collected from the study participants from their respective institutions after obtaining data collection permissions from the institutions’ authorities. Data were collected using a personal hard copy and also a web-based questionnaire. The data collection process occurred over a period of three months (from 10 October 2022 until 10 January 2023).

Statistical analysis

Data were analyzed using Statistical Product and Service Solutions (SPSS version 28) (IBM Corporation, Armonk, NY, USA). Descriptive statistics were calculated, and a chi-square test was used to determine the association of categorical variables (different levels of nicotine dependence - FTND) with gender and academic year. An independent sample t-test was used to compare the mean difference of continuous variables (nicotine dependence - KTSND) across genders, and one-way ANOVA was used to compare the mean difference among different academic year levels.

## Results

In the present study, 600 students from six dental institutions located in Riyadh, Saudi Arabia, were approached and requested to participate, out of which 430 dental students agreed to participate by providing written informed consent, with a response rate of 71.6%.

Demographic details of all 430 study participants were analyzed. Among these study participants, 70 (16.30%) participants were 24 years old, 137 (31.90%) were between 19 and 21 years of age, and 223 (51.90%) were between 22 and 24 years of age.

Two hundred six (47.90%) of the study participants were female, and 224 (52.10%) were male participants. Moreover, 65 (15.10%) participants were from Alfarabi University, 69 (16.00%) from Dar Aluloom University, 79 (18.40%) from King Saud Bin Abdulaziz University for Health Sciences, 66 (15.30%) from King Saud University, 64 (14.90%) from Princess Nourah University, and 87 (20.30%) from Riyadh Elm University. 

Thirty-nine (9.10%) study participants were in their first clinical year, 53 (12.30%) in the second clinical year, 56 (13.00%) in the third clinical year, 69 (16.00%) in the fourth clinical year, 95 (22.10%) in the fifth clinical year, and 118 (27.40%) in the sixth clinical year (Table 1).

**Table 1:** Demographic details of the study participants.

Demographic details	Parameters	Frequency	Percentage
Age	>24	70	16.30%
	19-21	137	31.90%
	22-24	223	51.90%
Gender	Female	206	47.90%
	Male	224	52.10%
College	Alfarabi University	65	15.10%
	Dar Aluloom University	69	16.00%
	King Saud bin Abdulaziz University for Health Sciences (KSAU-HS)	79	18.40%
	King Saud University (KSU)	66	15.30%
	Princess Nourah University	64	14.90%
	Riyadh Elm University	87	20.30%
Academic year	1^st^ clinical year	39	9.10%
	2^nd^ clinical year	53	12.30%
	3^rd^ clinical year	56	13.00%
	4^th^ clinical year	69	16.00%
	5^th^ clinical year	95	22.10%
	6^th^ clinical year	118	27.40%

In the present study, out of the 430 dental students who agreed to participate, only 120 participants were current tobacco smokers, and further data were analyzed only for these 120 participants. Out of these 120 (27.90%) smokers, the majority of them (71, 59.20%) initiated smoking after enrolling in the dental program (Table 2).

**Table 2:** Details of smoking habits.

Questions related to smoking habits		Frequency	Percentage
Do you smoke cigarettes?	Yes	120	27.90%
	No	310	72.10%
How many packs of cigarettes have you smoked in your lifetime?	5 or more packs/100 cigarettes	120	100.00%
When did you start smoking cigarettes?	After joining the dental program	71	59.20%
	Before joining the dental program	49	40.80%

The level of nicotine dependence was assessed using FTND. Twenty (16.66%) study participants displayed very low nicotine dependence, 40 (33.33%) displayed low nicotine dependence, 18 (15%) displayed moderate nicotine dependence, 24 (20.00%) study participants displayed high nicotine dependence, and 18 (15.00%) displayed very high nicotine dependence. When compared between different genders eight (25.80%) of the female participants displayed very low nicotine dependence, 12 (38.70%) displayed low nicotine dependence, four (12.90%) displayed moderate nicotine dependence, five (16.10%) of the female participants displayed high nicotine dependency, and two (6.50%) displayed very high nicotine dependency. Among the male participants, 12 (13.50%) of the male participants displayed very low nicotine dependence, 28 (31.50%) displayed low nicotine dependence, 14 (15.70%) displayed moderate nicotine dependence, 19 (21.30%) displayed high nicotine dependency, and 16 (18.00%) displayed very high nicotine dependency. Despite the fact that the male participants (88.90%) and students in their sixth clinical year demonstrated very high nicotine dependence in comparison to their peers, the statistical tests did not show a significant difference between the groups (P > 0.05) (Table 3).

**Table 3:** Comparison of nicotine dependence (FTND) between different genders and academic years.

Parameters		Very Low		Low		Moderate		High		Very High		χ2 Value	P-value
		N	%	N	%	N	%	N	%	N	%		
120 study participants		20	16.66%	40	33.33%	18	15.00%	24	20.00%	18	15.00%		
Gender	Female	8	25.80%	12	38.70%	4	12.90%	5	16.10%	2	6.50%	4.929	20.796
	Male	12	13.50%	28	31.50%	14	15.70%	19	21.30%	16	18.00%		
Academic year level	1st clinical year	1	16.70%	2	33.30%	2	33.30%	1	16.70%	0	0.00%	0.295	0.409
	2nd clinical year	1	7.70%	5	38.50%	2	15.40%	3	23.10%	2	15.40%		
	3rd clinical year	2	10.50%	11	57.90%	2	10.50%	2	10.50%	2	10.50%		
	4th clinical year	1	5.30%	10	52.60%	3	15.80%	2	10.50%	3	15.80%		
	5th clinical year	6	27.30%	4	18.20%	4	18.20%	5	22.70%	3	13.60%		
	6th clinical year	9	22.00%	8	19.50%	5	12.20%	11	26.80%	8	19.50%		

In the present study, the average KTSND score for nicotine dependency among dental students who smoke was found to be 1.814 ± 0.39, with a higher mean score of 2.49 ± 0.8 for "tobacco has effects to relieve stress" and a least score of 1.28 ± 1.04 for "tobacco is part of the culture," 1.29 ± 1.21 for "smoking itself is a disease," 1.99 ± 0.94 for "tobacco is one of life’s pleasures," 1.67 ± 0.94 for "smokers lifestyles may be respected," 1.70 ± 1.01 for "smoking sometimes enriches people’s life," 2.06 ± 0.99 for "tobacco has positive physical or mental effects," 1.98 ± 0.96 for "tobacco enhances the function of smokers’ brain," 1.54 ± 1.13 for "doctors exaggerate the ill effects of smoking," and 2.14 ± 0.92 for "people can smoke at places where ashtrays are available" (Table 4).

**Table 4:** Mean distribution of Kano Test for Social Nicotine Dependence (KTSND) score.

Questions	Mean	SD
Smoking itself is a disease	1.29	1.21
Smoking is a part of culture	1.28	1.04
Tobacco is one of life’s pleasures	1.99	0.94
Smokers lifestyles may be respected	1.67	0.94
Smoking sometimes enriches people’s life	1.70	1.01
Tobacco has positive physical or mental effects	2.06	0.99
Tobacco has effects to relieve stress	2.49	0.80
Tobacco enhances the function of smokers’ brain	1.98	0.96
Doctors exaggerate the ill effects of smoking	1.54	1.13
People can smoke at places where ashtrays are available	2.14	0.92

In the present study, the participants displayed high KTSND scores, male participants with a mean score of 18.2 ± 5.4 and female participants with a mean score of 17.97 ± 5.71. However, an independent sample t-test displayed no statistically significant difference in the total KTSND score between male and female participants (t=0.203; P=0.839). Similarly, to one-way ANOVA, the cumulative KTSND score across academic years showed no statistically significant difference (F = 1.815; P = 0.116) (Tables 5, 6).

**Table 5:** Comparison of the total KTSND score between different genders.

Gender	N	Mean	SD	t	P Value
Male	89	18.20	5.40	0.203	0.839
Female	31	17.97	5.71		

**Table 6:** Comparison of the total KTSND score with different academic years.

Academic Year	Total KTSND Score				
	N	Mean	SD	F	P Value
1st clinical year 2nd clinical year 3rd clinical year 4th clinical year 5th clinical year 6th clinical year	10	15.17	5.49	1.815	0.116
	13	18.77	4.19		
	19	17.63	6.34		
	19	21.16	3.91		
	20	17.60	5.62		
	38	17.42	5.65		

## Discussion

Dental professionals play a key role in integrating smoking prevention and cessation programs as a part of their oral health promotion activities [30,31]. They are well-positioned to provide health promotional activities, which can include tobacco prevention and cessation, as they frequently encounter these patients and have better access to identifying the effects of tobacco products on the patient’s oral cavities [31-35]. The World Health Organization also emphasizes on training and educating dental health professionals on early detection and diagnosis of adverse effects of tobacco and planning early intervention and treatment for the same [36]. However, health professionals who smoke may not be efficient and effective in tobacco cessation interventions [37,13]. Considering the role of dental professionals as role models for tobacco prevention activities, it is important to know their attitudes and behavior toward tobacco usage. In the present study, the prevalence of cigarette smoking among dental students was found to be 26.7%. These findings were less than those reported in India [11]. However, the prevalence rate was higher in comparison to Australian dental students and previously reported data among dental students in Saudi Arabia, where the authors had reported a prevalence of 17-22% among dental students [12-14]. Alotaibi et al., in a meta-analysis, reported that the pooled estimate of smoking prevalence among college students in Saudi Arabia was 17% [38]. Our findings were similar to a study conducted by Singh et al., where they reported on the usage of smoking and smokeless forms of tobacco among dental students in India and found a prevalence of 23.8%, with 44.5% and 32.8% prevalence for smoking and smokeless forms of tobacco, respectively [39]. Rodakowska et al. reported an even higher amount of prevalence of tobacco smoking among dental students from Poland and Italy, which was found to be 42% and 28%, respectively [40]. In the present study, we also found that 71 (59.20%) of the participants reported initiating smoking only after joining the dental program.

In the present study, nicotine dependence among dental students was assessed using FTND, which is designed to assess an individual’s physical dependence on nicotine [21,22]. A recent systematic review reported on the psychometric properties of FTND, where the authors assessed 11 articles that had assessed the test re-test reliability of FTND across different populations, and its internal consistency was assessed using Cronbach’s alpha. The average reliability score was 0.75 moderate test reliability, and Cronbach’s alpha coefficient ranged from 0.70 to 0.90, indicating a good internal consistency [41].

In the present study, out of 430 study participants, only 120 were smokers, and further data were analyzed only for these 120 participants. Five (16.10%) of the female participants displayed high nicotine dependency, and two (6.50%) displayed very high nicotine dependency. Among the male participants, 19 (21.30%) displayed high nicotine dependency, and 16 (18.00%) displayed very high nicotine dependency. These findings were higher than the level of nicotine dependence reported among medical students in India as reported by Bhujade et al., where only four (1.8%) were found to have high nicotine dependency [9]. Demüralay et al. also reported 15 (24.6%) having high nicotine dependence and six (9.8%) very high dependence among medical students in Turkey [7]. In the present study, we also assessed the social nicotine dependence using KTSND [27,28]. It mainly deals with assessing the misperception of smoking tobacco products among smokers, for example, denying the harmful effects of tobacco smoking or justifying themselves by stating that smoking is a part of their social and cultural behavior [28,42]. It comprises 10 items: “smoking is a disease in itself”, “smoking is a part of the culture”, “smoking is one of life’s pleasures”, “a smoking lifestyle should be respected”, “some people’s lives are enriched by smoking”, “smoking has physical or mental benefits”, “a cigarette is a stress reliever”, “cigarettes enhance the function of smokers’ brains”, “doctors exaggerate the harm of smoking”, and “a place with an ashtray is a place". This tool is designed to assess the psychological aspects of smoking [27,29]. In the present study, the study participants reported high KTSND scores, male participants with a mean score of 18.20 and female participants with a mean score of 17.97. However, the independent sample t-test displayed no statistically significant difference in the total KTSND score between male and female participants. The KTSND score reported in our study was higher than other studies reported in the literature. Huang et al. reported a mean KTSND score of 11.6 among dental students in Australia [12]. Didilescu et al. also reported a mean KTSND score of 14.1 among dental students in Romania, and they also reported that the female participants displayed higher social nicotine dependence in comparison with the male participants [43]. Even though the KSTND scale was originally developed in Japan and its psychometrics properties assessed earlier in previous studies were also reported among the Japanese population, the validity of this scale on the Saudi Arabian culture is questionable as the cultural adaptation may vary among different populations. This could be one of the limitations of this study.

These findings of social nicotine dependence are important, as they form the misperception of smoking among smokers, such as justifying smoking as culturally acceptable or smoking as a common social behavior. Reports also suggest that there is a correlation between social and physical nicotine dependence [28]. Considering the fact that nicotine dependence is one of the main barriers to successful tobacco quit attempts [44], dental students need to be educated and motivated to attend smoking cessation interventions, in order to reduce their nicotine dependence, along with adjunctive nicotine therapy, which has proven to be effective in managing nicotine dependence. Supportive environments favorable for treatment should be created in health universities [45,46]. A recent study conducted by Sharanesha et al. reported on the awareness and perception of dental students toward nicotine replacement therapy (NRT) in Riyadh, Saudi Arabia, where they found that 74% of the study participants were aware of NRT. However, 50% of them considered that motivating smokers on NRT was a waste of time, and 65% considered it difficult to quit smoking [47]. Similar findings were reported by Khalaf et al., where the majority of dental students from Kuwait were willing to brief their patients on smoking cessation, but 62% perceived their role that smoking cessation was difficult [48]. However, Gaidhankar et al. reported on the awareness and positive attitude of dental students in India toward NRT and tobacco counseling [49]. 

In this study, the average KTSND score for nicotine dependency among dental students who smoke was found to be 1.814 ± 0.39 with a higher mean score of 2.49 ± 0.8 for “tobacco has effects to relieve stress,” which states that the majority of participants considered tobacco to have a stress-relieving effect. These findings were consistent with the results of the studies reported in the literature. AlSwuailem et al., in their study on smoking among dental students in Saudi Arabia, reported that 47.8% of the study participants considered stress as a main factor for smoking cigarettes [13]. Alrasi et al., in their study on smoking prevalence among health professional students in Saudi Arabia, reported that 33.3% of the dental students in their study reported that smoking was helpful for them in relieving their stress and 41.7% considered smoking as an enjoyment during leisure time [50]. Alsaad et al., in their study on the prevalence of tobacco smoking among dental practitioners in Saudi Arabia, reported that 52.6% of the study participants considered stress relief as a main reason for smoking, and 31.6% considered smoking as a way for relaxation [51].

In the present study, out of 120 study participants who were smokers, the majority 71 (59.20%) reported initiating smoking only after joining the dental program. These findings were in accordance with the results of the study conducted by Mansour, where he reported that more than 50% of dental students in Jeddah, Saudi Arabia, initiated smoking only after joining the dental program [14]. Hence, these findings are quite alarming and need to be addressed with priority.

Considering the harmful effects of tobacco products on an individual’s health and well-being and knowing the vital role of dental professionals in tobacco prevention and cessation interventions, it is important to design and implement tobacco control programs for dental students. Studies have reported a decrease in social nicotine dependence among dental students after attending tobacco control educational programs in their health universities [52]. A country like Saudi Arabia, an Islamic nation, considers smoking a sinful practice; hence, smoking among health professional students is a crucial concern [53]. Considering the health aspects of the young generation of doctors and their role in society as facilitators of tobacco cessation interventions, there is a need to develop education activities coupled with professional counseling and pharmacological treatments. Dental students’ perception of tobacco cessation intervention needs to be assessed in order to achieve successful quit attempts. Maharani et al. [54] and Priya et al. [55], in their studies, have highlighted the need for developing a dental curriculum promoting professional responsibility toward tobacco cessation intervention. This can enhance the dental students’ knowledge and confidence toward involvement in tobacco cessation interventions for their patients [54,55]. Dental students' perceived barriers toward involvement in tobacco cessation interventions in dental institutions also need to be considered. These mainly include dental students themselves using tobacco products, lack of confidence, considering dental clinics not suitable for counseling, and believing themselves incompetent for engaging patients in tobacco cessation intervention [55,56]. Smoking cessation interventions at a younger age limit the health risks associated with smoking. Hence, antismoking programs and policies should be developed and implemented in health professional institutions. Considering the previously published reports on higher levels of stress and anxiety among dental students during their education in Saudi Arabia [57], and as a known risk factor for adopting adverse habits like smoking, it is important to plan activities that aim at stress management. Students undergoing stress should be encouraged to adopt healthy lifestyles, which include healthy eating, regular exercise and sports activities, adequate sleep, yoga, and meditation [58,59]. The dental education system has an important role to play in developing dental curricula with emphasis on the role of dental students as facilitators for the prevention of tobacco usage and tobacco cessation interventions for their patients. Alhussain et al. reported on the perception of dental students toward smoking cessation counseling in Riyadh, Saudi Arabia, where 72.9% of the study participants displayed interest in getting trained to counsel their patients to quit smoking. However, 67% of the study participants considered a lack of training on smoking cessation and counseling as a barrier to helping patients quit smoking [60]. The current dental and medical education in many developed countries like the United States and Canada merely emphasizes knowledge-based tobacco education in their curriculum [61,62]. There is also a considerable amount of variation in the attitude of dental students toward smoking cessation programs as there is a lack of formal training in the dental education system [63]. Emphasis should be given to developing policies and making changes in the dental curriculum by incorporating tobacco cessation counseling/intervention training programs in dental education. Dental schools should also emphasize more on training and developing skills by actively engaging dental students in tobacco prevention and cessation interventions for their patients [64,65].

In the present study, social desirability bias could affect the reporting of smoking prevalence among dental students, which could be a limitation in this study. The second limitation is that, in this study, we considered only combustible cigarette usage and its nicotine dependence. The third limitation was comparing our findings with those of medical students since there were very limited studies that reported nicotine dependence among dental students. The fourth limitation was with the cultural adaptability of the KTSND tool for the Saudi Arabian population, which was not assessed in this study.

## Conclusions

Dental students, despite being the facilitators in tobacco prevention and cessation programs, have displayed a high prevalence of cigarette smoking. The majority of the participants reported initiating smoking only after joining the dental program. The physical dependence on nicotine was assessed using FTND, and the social nicotine dependence was assessed using KTSND. The majority of the participants considered tobacco to help in relieving stress. The findings of this study should be considered for designing specific tobacco cessation programs for dental students along with effective stress management programs. Future studies should consider the limitations of this study, especially with the sampling error, and cultural adaptability of the tools and should focus on all forms of smoking devices. Reforms need to be made in the dental curriculum, and dental students should be oriented and trained in their institutions to recognize their responsibilities in tobacco cessation interventions. Stringent policies need to be developed and implemented in order to bring down tobacco usage among health professional students.
